# Music Listening as Coping Behavior: From Reactive Response to Sense-Making

**DOI:** 10.3390/bs10070119

**Published:** 2020-07-20

**Authors:** Mark Reybrouck, Piotr Podlipniak, David Welch

**Affiliations:** 1 Musicology Research Group, Faculty of Arts, KU Leuven-University of Leuven, 3000 Leuven, Belgium; 2 IPEM, Department of Art History, Musicology and Theatre Studies, 9000 Ghent, Belgium; 3 Institute of Musicology, Adam Mickiewicz University in Poznań, 61–712 Poznań, Poland; podlip@poczta.onet.pl; 4 Institute Audiology Section, School of Population Health, University of Auckland, 2011 Auckland, New Zealand; d.welch@auckland.ac.nz

**Keywords:** music listening, music as environment, coping behavior, phylogenetic constraints, sensory modalities, sense-making, adaptive behavior, addiction

## Abstract

Coping is a survival mechanism of living organisms. It is not merely reactive, but also involves making sense of the environment by rendering sensory information into percepts that have meaning in the context of an organism’s cognitions. Music listening, on the other hand, is a complex task that embraces sensory, physiological, behavioral, and cognitive levels of processing. Being both a dispositional process that relies on our evolutionary toolkit for coping with the world and a more elaborated skill for sense-making, it goes beyond primitive action–reaction couplings by the introduction of higher-order intermediary variables between sensory input and effector reactions. Consideration of music-listening from the perspective of coping treats music as a sound environment and listening as a process that involves exploration of this environment as well as interactions with the sounds. Several issues are considered in this regard such as the conception of music as a possible stressor, the role of adaptive listening, the relation between coping and reward, the importance of self-regulation strategies in the selection of music, and the instrumental meaning of music in the sense that it can be used to modify the internal and external environment of the listener.

## 1. Introduction

Coping is a survival mechanism of living organisms in their interaction with the environment. As a broad category, it has been defined as the “cognitive and behavioral efforts to manage specific external and/or internal demands that are appraised as taxing or exceeding the resources of the person” [1] (p. 141). Coping, in this view, is not only reactive behavior, but also involves making sense of the environment, ranging from overt physical reactions to mental and cognitive operations. As evolved biological beings, we have a dispositional heritage that prepares us for survival, and even though the conditions in our technological society have changed drastically, our biology has preserved the capacity for coping in threatening environments. 

Arguing on these lines, we theorize that coping mechanisms contribute to our experience of music. Music listening can be very challenging, even so challenging that listeners are not able to make sense of it. The challenge, moreover, can be physical, as in cases of loud music, but also intellectual, as in cases of complex and sophisticated music. This article aims to provide an overview of the evolutionary and ecological underpinnings of coping as applied to listening to music [2]. We also challenge traditional approaches to musical sense-making that conceive of music as a structure that can be analyzed at an abstract and detached level of processing outside of the time of actual unfolding. Rather, we propose a “back-to-basics” approach that conceives of music as a succession of acoustical events that impinge upon the body and the mind. This is a naturalistic approach to musical sense-making that argues for a continuity between environmental sounds and music and draws upon the seminal ideas of pragmatic philosophers such as Dewey and James with their repeated emphasis on “having an experience” (see [2,3] for an overview).

We hope this theoretical work will encourage future research and theory-development in the study of music in three ways: (i) by redefining the process of musical sense-making in terms of coping behavior; (ii) to open the definition of coping beyond being conceived merely as a tool for survival into its role as a tool for (musical) sense-making, and (iii) to revalue the concept of adaptive and maladaptive listening as a way of coping with musical sounds. There is, in fact, a whole area of research that deals with music in terms of coping strategies, particularly in the context of coping with stress [4,5,6,7]. Our theoretical proposal rests on two assumptions: music is vibrational and transferable energy that impinges upon our senses; and music can be seen as a challenging environment, both in a positive or negative sense. Starting from these assumptions, we argue for two additional claims that much can be learned from the evolutionary and ecological underpinnings of coping strategies, both in general and as applied to music listening; and it is possible to conceive of music as a stressor or reward with the related concepts of adaptive listening in search of reward and maladaptive listening in search of arousal.

## 2. Music as Sound Environment: Natural and Man-Made Sounds

Coping behavior, in a common but narrow definition, is a survival mechanism to avoid life-threatening conditions in the environment. This environment may be threatening or advantageous, and the extent to which an organism can assess the outcome of a possible encounter may influence its survival. Human beings, as biological organisms, are equipped with a hereditary dispositional machinery, consisting of sensory organs, motor tools (the musculoskeletal system), and a central processing system, in a body that sustains itself through many interacting physiological processes that contribute to maintaining a state of equilibrium with regard to the internal and external environment. This equilibrium is commonly referred to as *homeostasis* [8], as the driving mechanism for self-regulation in the nervous system [9]. Its maintenance depends on the senses that detect internal and environmental changes in the conditions for optimal functioning—including many variables such as body temperature and body fluid balance, which should be kept within pre-set limits—and which allow us to address these changes via internal adaptive mechanisms and interactions with the external environment. This holds, in particular, for the sense of hearing, which, from a biological point of view, may be considered to be an acoustic warning system [10,11], which primary function, like that of other senses, is to recognize the energy changes in the environment. Acoustic cues, accordingly, are interpreted in terms of optimal navigation in the environment for survival and reproduction [12].

**Figure 1:**
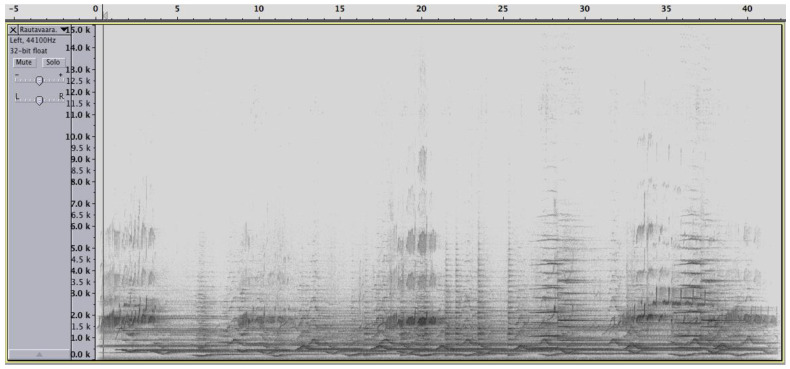
Spectrogram of the beginning (starting at measure 24) of Rautavaara’s Cantus Arcticus. The sounds of the symphonic orchestra are intermingled with bird calls, with typical recognizable and repeated visual patterns.

Music, in this view, is a subset of the broader sonic universe. It can be considered as a *sound environment*, which encompasses both natural and man-made sounds, which can be used as music, considered separately or in combination. This is illustrated in Figure 1, which gives an example of a musical/sonic environment, which is a hybrid combination of both natural and man-made sounds. It shows a short excerpt of Rautavaara’s Concerto for Birds and Orchestra (Cantus Arcticus). The spectrogram shows the combination of the sounds of a symphonic orchestra, with a rich spectral content mostly between 200 and 7000 Hz and bird calls with a very specific and recognizable spectral content and temporal course. Even a cursory inspection of the spectrogram shows spectral patterns that function as figures against a ground. This is quite obvious at 8–11 s, at 18–20 s, and again at 32–34, 34–37 s, and 39–42 s. There are also orchestral sound combinations with high recognizability at 6–7 s and at 21, 22, 23 and 25 s (sustained tones with a melodic turn) and a beautiful dynamic curve from 26 to 30 s. 

Environmental and/or musical sounds can be examined both in an objective and subjective way [13]. There is, however, no absolutely objective analysis by the ear because the outer ear introduces distortions to incoming sounds, which preferentially respond to spectra relevant to survival [14], as do the middle and inner ear. Furthermore, the functioning of the Organ of Corti is influenced by the central nervous system via efferent neuronal tracts, which also influence the function of the cochlear amplifier [15,16].

Hearing, moreover, allows us to interpret sounds on the basis of their spectral content, temporal information, and sound level, with maximum sensitivity to the spectral information from 1200 to 2400 Hz [17,18]. There is a further distinction between natural and man-made acoustic cues. Music is man-made, and the history of musical instrument building shows an abundance of explorations of sounding materials (wood, stone, copper, gut strings, membranes, vibrating air columns) and ways of sound generation by applying physical forces on these materials (bowing, blowing, hitting, striking, plucking, rubbing, etc.) [19,20,21]. The techniques of sound generation and the materials used have been selected with the aim to produce specific and typical dynamic ranges, crest factors, fundamental frequencies, modifications of the ADSR curve—a common type of musical envelope that describes how a sound changes over time, consisting of Attack, Decay, Sustain, and Release—and spectral distortions of all kinds. This holds for traditional acoustic instruments, but it holds *a fortiori* for the unrestricted possibilities of electronic modification and amplification of the sounds.

Just as vocal systems can be overblown, the physical hardware of amplification systems can also be used to emulate high arousal. The principle is quite simple: by applying an amplitude gain accompanied by a low-pass filter, the signal is pushed toward a saturation point with nonlinear alterations as a result. This nonlinearity is then filtered again to generate an output that becomes a multi-band-passed nonlinearity [22]. Listeners, as a rule, display psychophysiological responses to such nonlinearities with measurable autonomic reactions, even when they are not deliberately aware of them [23].

**Figure 2:**
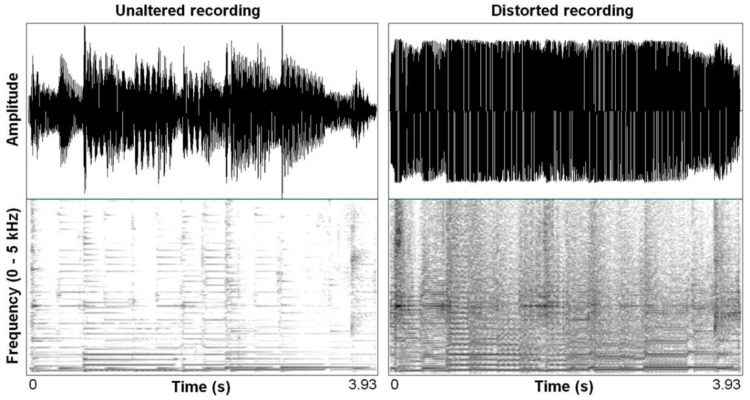
Waveform and spectrogram (Fast Fourier Transform (FFT) method, window length 0.005 s; Gaussian window shape, dynamic range 50 dB) of an acoustic guitar melody, unaltered (**left**) and distorted (**right**) using a wave shaping function (Camel Crusher VSY Plug-in). Reproduced without modification from Bryant, 2013. (Creative Commons Attribution Licence).

**Figure 3:**
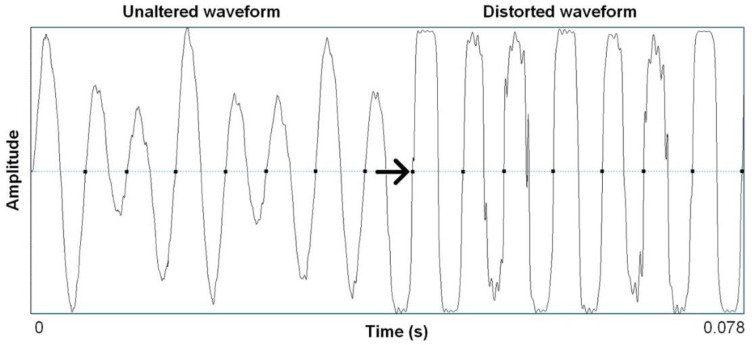
Waveform segments from the recording in Figure 2 of seven cycles (78 ms) of unaltered acoustic guitar and the same seven cycles after wave shaping functions (Camel Crusher VST Plug-in). Reproduced without modification from Bryant, 2013. (Creative Commons Attribution Licence).

Figure 2 and Figure 3 provide an example of such modification of a natural acoustic sound (guitar) so as to produce a “hot” sound, which is characterized by a greater density of the spectral contents of the sounds and more rectangular waveforms with the aim to produce an overblown amplification effect. They exemplify quite clearly how distortion effects, which are so commonly used in contemporary music, most typically in early garage rock and punk rock, mimic in important ways the nonlinear characteristics that are also seen in highly aroused animal signals and human voices. As humans, we have a sensitivity to such features that is rooted in a highly conserved mammalian vocal signaling system. Animal excitement, in fact, is characterized by physiological activation that prepares the animal for immediate action (fight or flight) and motivates vocal communication to affect the behavior of others in an urgent way. As such, animals produce shrieks, alarm calls, and contact calls that demand particular responses, both perceptually and behaviorally [24]. They produce sound waves that reach their maximal amplitude, which correlate perceptually with a harsh, noisy sound that penetrates noisy environments and that is hard to habituate to, which is typical of conspicuous signals that require a quick response or attention [25].

## 3. Music Listening as Coping Behavior

Stress is a responsive behavior that consists of three processes: primary appraisal, secondary appraisal, and coping [26,27,28]. Primary appraisal refers to the process of perceiving something as a threat; secondary appraisal brings to mind a potential response to that threat; and coping is the process of executing that response. Coping can be positive or negative, dependent on the appraisal of stimuli as opportunities or threats [29,30,31] and can be adaptive or maladaptive in the sense that the outcome is emotional adjustment or physical health [32] (see below). The positive approach involves exploring and benefitting from the opportunities afforded by the environment; if the environmental stimuli are threatening, however, the coping may become negative, revolving around avoidance of harmful aspects of the environment. Part of coping may thus be semiotic, involving an aspect of sense-making where appropriate coping techniques depend on the meaning of cues that lead to negative or positive estimations. 

The meaning—and thus the type of coping—depends on the power human beings have over their environment [33,34,35,36]. This power is characterized by four dimensions: awareness, choices, freedom to act intentionally, and involvement in creating change. To be powerful, in this view, is to be able to exercise one’s capacity to participate in creating what is happening by bringing about specific ongoing changes, which are nonrepeating and increasingly diverse. Humans, then, can use power to freely choose with the awareness to create changes.

When applied to music, the role of power and coping is apparent. Music is pervasive and ubiquitous in urban environments. It can be heard in public spaces, restaurants and bars, fitness centers, and even in the private sphere of an apartment if the neighbor does not care about loudness restrictions. It is not possible to “close” our ears in order not to hear the music that we do not want to hear. Music, as such, can be annoying because it intrudes into the private sphere and can even become a kind of “acoustic violation,” given the penetrating power of loud sounds, if music is considered from the acoustic-vibrational point of view. Much depends here, however, on our subjective evaluation and the feeling of power [37,38,39,40,41]. There are, as such, both subjective and objective criteria to perceive music as threatening (see [39]). Music, then, can be a stressor for those who hear it, so people may need to cope.

Music can be perceived both as a threat or an opportunity, depending on the perspective taken by each listener. Coping behavior should thus be conceived not merely as a tool for survival, but also as a tool for (musical) sense-making, or stated in other terms: musical sense-making can rely on coping strategies as one of its underlying explanatory mechanisms. This implies that power is considered as the capacity “to participate knowingly in the nature of change characterizing the continuous patterning of the human and environmental fields” [42] (p. 9). Translated to the realm of music, this is related to the active process of sense-making, either by exerting physical interactions on sound-producing devices, in the case of playing a music instrument, or epistemic interactions on actual sounds or mental replicas of the sounds in case of listening to and reflecting on the music. The latter involves mental operations that are at work in knowledge construction such as exploring, observing, measuring, labeling, selecting, comparing, recognizing, analyzing, interpreting, considering, choosing, reproducing, and modifying, etc. (see [43] for an overview). 

## 4. Coping and Sense-Making 

Our senses allow us to experience qualitative aspects of the world. Conscious “sensations” provide the first level; they are explicitly connected with the senses but as sensations, they are not necessarily related to a cause or object. This is the case, in contrast, with conscious “perception”, which is typically integrated into a consciously perceived three-dimensional world. According to Gamez [44] a “conscious sensation” refers to the awareness of an individual aspect of sensation (e.g., a color, sound, smell, sudden noise, pain, or buzzing on the skin, etc.), whereas “conscious perception” refers to the integration of many conscious sensations to create a perceptual object. This transition from sensation to perception can be very smooth, as in the case of a sudden noise, where we are first startled by the noise that swamps our senses, but some moments later, we are able to locate the source and nature of the sound. What matters, in this regard, is the relationship between our consciousness and the physical world, with a possible transition from mere sensory stimulation to the allocation of meaning and semantic weight.

**Figure 4:**
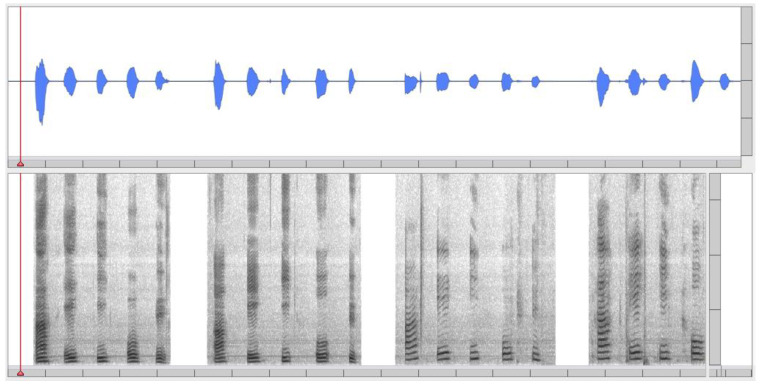
Analog-continuous depiction of the vowels /ɑ/ /e/ /ɪ/ /o/ /ü/ both as a waveform (upper pane) and a spectrogram (lower pane): two male voices (left) and two female voices (right).

Making sense of music, accordingly, holds a dynamic tension between ongoing acoustic stimulation and the way we cope with it in terms of perceptual categories. It proceeds either in real time with the sensory stimuli impinging in a direct way on the senses (in-time processing), or in a more detached way that takes more distance with respect to the actual unfolding (outside-of-time processing) [45,46,47]. The real-time experience is mainly continuous as it keeps track of the acoustic flux, which unfolds over time. In this case, it works outside of the limitations of a relatively small set of discrete categories for pitch and note durations, as exemplified most typically in a score with its reliance on symbolic notation, so as to be more sensitive to ranges and transitions that are continuous rather than discrete. As such, it is *analog* rather than *digital* in the sense of von Neumann’s original conception of computing automata, which he classified as either “analogy machines” and “digital machines.” The former are based on the principle that numbers are represented by certain physical “quantities,” such as the intensity of an electrical current or the size of an electrical potential; the latter work with the familiar method of representing numbers as aggregates of “digits” as used when we proceed with individual, non-mechanical computing by using integers [48] (pp. 292–294; see [43] for the analog/digital and continuous/discrete analogy). The outside-of-time experience, on the contrary, reduces the temporal unfolding to single representations or associations that work at a virtual level of imagery and that represent the sonic world without actual coupling with the minutiae of the particular temporal and spectral unfolding of the sounding music. They mostly act at a symbolic level of representation and computation and are basically “rate-independent”, which means that the time of measurement or assigning meaning to them has no coherence with the time of the dynamics of the unfolding. The computations, in this view, are free of all influences other than the internal mental operations [49]. Such symbolic representations have the advantage of “distinctness” and “communicability” by stressing the economy of abstraction rather than the richness and subtlety of experience. They are exemplified most typically by using numbers or letters or words, hence the term digital-discrete, and basically mean that something can be apprehended in an all-or-none fashion, thus featuring its distinctive features rather than the particularities of a typical rendering. Speaking or thinking in terms of the sound of “*a* flute” or “*a* major chord” in the abstract, as against the sound of “*this* flute” or “*this* major chord”, as they actually sound, can make this clear. Figure 4 provides a rather trivial example. It depicts an analog-continuous depiction of the vowels /ɑ/ /e/ /ɪ/ /o/ /ü/ in four different renderings (two male and two female voices). The vowels of each rendering are four phonemes that are the same and that can be symbolically represented as discrete letters of the alphabet; their spectrograms, in contrast, are different and can be considered as an analog depiction of these phonemes. 

The distinction is of paramount importance for the process of coping with the sounds. The same sound, in its phonetic or symbolic transcription, can convey a totally different meaning, depending on its acoustic and spectrographic cues. This is also exemplified in animal calls such as alarm calls, attraction and communication calls as well as in preverbal vocal utterances of newborns, in the prosodic features of human talk and to a major extent also in human singing (see [39]). It is thus possible to conceive of acoustic patterns in two distinct ways: one is to focus on the minute and particular acoustic cues; the other is the reduction of these cues to the more encompassing global auditory event. This is the distinction between *everyday listening* and *musical* or *acoustical listening* as the terms are used in the context of ecological perception [50,51]. Everyday listening reduces the incoming stimuli to the perception of sounding events, the latter examines the sound with respect to its acoustic qualities. There is, as such, a distinction between what we actually hear and the interpretation or valuation of what we hear. What matters in this approach is a whole machinery of assigning semantic meaning to the surrounding world, reducing its complexity to major categories. Or to put it differently: what we are listening to are not sounds, but signs that shape our world [52,53]. Listening, then, can be considered as a search for information, which means that we do not perceive the environment primarily in terms of its objective descriptions, but in terms of sequences of stimuli or *ecological events* that activate our coping mechanisms [54,55,56].

We should be careful, however, not to generalize too quickly from event perception to perception in terms of discrete categories. What common listeners experience is mostly the experience of a kind of *dynamic feeling*, which is different from specific, conceptual assignments. This means that the living experience of music, as an internal experience of shifting and conflicting responses to our environment is cognitively and biologically different from *propositional* or *verbal statements* about the music, in the sense that it depends first of all of the same subcortical structures that trigger our fight-and-flight responses. This has been shown convincingly in the case of emotional responses to music, but it also holds for the processing of complex information such as musical structure [57]. As such, there seems to be a dynamic tension between lower-level non-conceptual and higher-level conceptual processing. This means that a conscious brain must use the sensory input to its body, but it must also be aware of its use. This is what Damasio calls the “feeling of what happens”, which he considers to be a basic requirement for conscious thought [58]. However, there is also the higher level, which brings us to the distinction Cassirer has drawn between animal and human cognition in general, and which can be reduced to the transition from a less solidified state of meaning—a state of liquefaction and diffused qualities—to a knowledge of definite, steadfast, and permanent objects [59] (p. 168). Crucial in this distinction is the role of language as a unique tool to fixate, concretize, and cognize objects in the world. This is, to some extent, the *logocentric demand* for an objective and hermeneutical representation, which uses verbal or symbolic labels (logocentric or logogenic, see [60], for an extensive discussion) to refer to things that are considered meaningful. It is a widely used strategy of interpretation that uses verbal concepts as tools for sense-making and that conceives of “something as something”—making it possible to comment with a verbal or propositional statement— as advocated by philosophers like Cassirer and Heidegger (see [61] for an overview). This representational approach, however, fails to deal with the world as experienced immediately without the mediation of linguistic tools. The latter are constrained in the sense that the linguistic-semantic categories of sense-making are only a subset of possible conceptual categories. Linguistic-semantic categories are systematically highlighted, foregrounded, isolated, and digitalized for the purpose of linguistic communication, and determine a language’s “expressive envelope”, i.e, which meaning and meaning combinations can or cannot be expressed in language [62]. As such, languages force an obligatory and discrete choice on language users, which they do not necessarily make when they conceptualize. There is a need, therefore, of ways of sense-making that go beyond the constraints of propositional and verbal thinking. It is not enough, in this view, to refer to music in terms of propositions, which assign a predicate to something (e.g., this is the sound of a clarinet; this is a g-minor chord; this is a cadence, etc.). What is needed, on the contrary, is a way of sense-making that is ongoing and continuous, in the sense that it keeps pace with the temporal unfolding of the sounds.

## 5. Neural Correlates of Coping with Sounds: Reactive Behavior and Phylogenetic Constraints

Organisms are born with a biological disposition for coping with the world. This is obvious in evolved survival-related behavioral reactions to sudden changes in signal intensity from the environment. Sudden increases in stimulus intensity, as a rule, are more meaningful than those involving a decrease in intensity, in the sense that a heightened or increasing sound level tends to indicate that something is near or approaching. This is advantageous for effective functioning and coping with the world [11,63].

Three major reactive mechanisms have been studied in this regard: the orienting response or orienting reflex; the acoustic startle response or startle reflex; and myogenic vestibular responses which, from an evolutionary-ecological point of view, may be associated with potential threats and opportunities.

The *orienting response* is a kind of involvement behavior that causes increased attention to stimuli in the environment. It can be defined as a behavioral response to a novel, sudden, and significant stimulus such as turning one’s head toward an unexpected noise, simply acting as a trigger for further conscious or unconscious assessment of a situation [64,65] (see also [63,66,67]. The *acoustic startle*, on the other hand, has received a lot of attention as it lends itself very well to quantitative analysis [68,69,70,71,72]. Being defined as a fast twitch of facial and body muscles evoked by a sudden and intense acoustic, tactile, or visual stimulus, it consists of eyelid closure and contraction of the facial, neck, and skeletal muscles of the body together with an arrest of ongoing behavior and changes in some autonomic functions such as heart rate. It is suggestive mainly of a protective function against injury from a potential predator or blow and prepares for fight and flight, and despite its simple design, it can be modulated by a number of external and internal variables [70]. In order to elicit a startle response, however, the stimuli must be sudden and intense (e.g., 90 dB increase within 12 milliseconds of onset for acoustic stimuli). Both the intensity and the duration of the stimulus, moreover, can act of modulating factors [69]. The acoustically evoked saccular *myogenic response*, on the other hand, is related to but distinct from the startle reflex, which, in general, is characterized by rapid habituation, a prolonged refractory period, and a longer latency than the sound-evoked myogenic responses [73,74]. The myogenic responses, in particular, have implications for human responses to loud sounds. Our inner ear combines the organs of balance (the vestibular system, consisting of the three semicircular canals, the utricle, and the saccule) and the auditory organ (the cochlea), but from an evolutionary point of view, the cochlea has come to replace the saccule as the primary organ of hearing. There is, however, some evidence, that the saccule has retained some acoustic function in higher vertebrates and that it is still sensitive to sound, mediating evoked myogenic responses to acoustic stimuli [75]. This saccular sensitivity, moreover, also has other consequences, as projections from the vestibular system to the autonomic nervous system have been documented at several levels of the nervous system [76,77]. Some of these consequences are affectively-laden, with several effects from nausea and changes in cardiovascular activity to hedonic responses associated with vestibular self-stimulation. They may be evoked by a range of natural stimuli such as voice intonation in speech, singing, crowd actions, and percussive music, all of which may have powerful affective associations.

**Figure 5:**
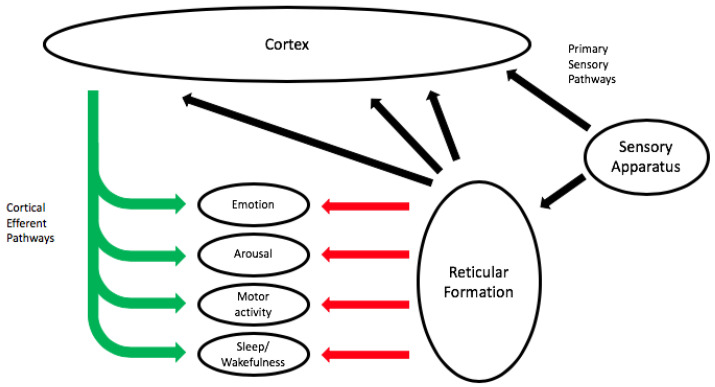
Schematic diagram of the reticular activating system including links between the sensory systems, the reticular formation, the cortex, and systems mediating physiology/behaviors that are activated by the reticular formation but are also under efferent control from the cortex.

There is further variability in reactive behavior related to habituation, sensitization, prepulse inhibition, and modification by prior associative learning [78]. *Habituation* involves a decrease in the magnitude of the behavioral response to iterative stimuli, enabling an organism to ignore stimuli that are considered to be irrelevant, thus minimizing the waste of energy. *Sensitization* involves an increase in the magnitude of the behavioral response, or a lowering of the response threshold upon repeated stimulations of the same type. Habituation, moreover, has been shown to be very important in music listening, particularly in the case of loud music, as the auditory system is highly adaptive to high-level sound with physiological adaptation occurring at multiple sites in the cochlea [79] and in the cortex [80]. This explains to some extent the policy of club managers to raise the sound levels through the course of the evening, starting mainly at 85 dBA Leq and reaching levels of around 97 dBA Leq later on, so as to meet the so-called wishes of customers for loud sound [40,81]. One of the underlying mechanisms is the search for arousal and excitement by stimulation of brainstem mechanisms such as the *reticular formation* or *reticular activating system* (RAS), as seen in Figure 5.

This RAS is a distributed network in the brainstem that modulates arousal in general and which is also involved in other sensory systems, in the initiation and control of motor activity, in autonomic arousal, sleep, and wakefulness, and emotions [82] and [83] (pp. 407–426). It can be considered as a major part of our phylogenetic disposition, as a kind of ancestral legacy, which can be considered as an inherited evolutionary old mechanism that allows us to cope with our environment, but which also has a major role in our valuation and reactions to sound.

The brainstem pathways and nuclei that process sound connect directly to the RAS, which operates in parallel and in interaction with these pathways and nuclei to modulate our experience of sound. It is also the source of the acoustic startle reflex, which is innervated by nerve cells that are located at early levels in the auditory pathway [71]. However, we are not startled every time we are exposed to loud sound, due to the mechanism of adaptation, and efferent control can also limit our startle responses. The brainstem reactions, moreover, are also closely related to early emotional reactions and are also coupled to some extent with feelings of tension and affect.

## 6. Musical Aesthetics and Coping Behavior: The Role of Adaptive Listening

As discussed above (Section 3), coping involves dealing with both threats and opportunities in the environment. In the experience of listening to music, the aesthetic qualities may depend on exploratory behavior in a changeable environment and the perception of environmental opportunities in the musical sound environment.

### 6.1. Exploration of the Sound Environment

Curiosity and exploitation of unexplored situational phenomena are important behavioral strategies that allow animals to compete for resources to survive and reproduce. The study of human responses to landscapes is a prototypical example in this regard: it involves the selection of places to live, the choice of habitats, emotional responses to key features of the environment, positive or negative feelings that motivate acceptance or withdrawal, exploration of the settlement and correlations with survival and reproductive success [11]. It has been shown that aesthetic responses to our environments relate to this ability to benefit from our environment. Humans prefer environments that facilitate and encourage exploration, wayfinding, and information gathering, and which signal the presence of resources that are necessary for survival. The savannas of tropical Africa, for example, appear beautiful to most people: they contain all that we need to survive such as easily obtainable nutritious food, trees to protect humans against the sun and that can be climbed to avoid predators, and long and unimpeded views that allow them to orient in space [84].

Music can be thought of in similar terms as a *sound environment* or *soundscape* with acoustic cues that are perceived as either beneficial or harmful [13]. There are objective characteristics that qualify certain types of sound as having positive or negative effects on our physical and mental health (see [39] for a broad overview). On the negative side, many studies have been conducted on the effect of high-level sound exposure during work and non-work or leisure activities such as music listening [10,17,38,85,86,87], and the possible damaging effect of high-level sound and noise has been demonstrated in a causal way. Lower-level sounds that are regarded as “noise,” however, do not necessarily cause injury, but may be annoying and have an impact on health via stress mechanisms [17]. On the positive side, some sounds are regarded by many people as pleasant such as waterfalls, mountain rivers, rain in the woods, the blowing of the wind, ocean waves, and many others, but particularly sounds of nature. These sounds, sometimes coined as “nature’s white noise”, are valued mainly for their relaxing and calming effect [88]. 

Besides the physical characteristics of the sounds, the listener’s perception of the sounds and their sources seem to play a major role in the valuation of the environment. This is obvious in *soundscape* studies, which explore the internal representation of the sound environment and a person’s emotional response to it [89]. A pair of soundscape dimensions called *pleasantness* and *eventfulness* has been proposed in this regard (e.g., [90,91]. This dimensionality is supported by an evolutionary theory [92,93] that states that the estimation of an environment as pleasant or unpleasant and the presence of many or few new events determines whether an environment is “rich” (pleasant and eventful), “dangerous” (unpleasant and eventful), “calming” (pleasant and uneventful), or “boring” (unpleasant and uneventful). These two dimensions reflect the basic dimensions of human mood, as expressed in earlier research [94] but the dimensionality of the soundscape may also be more complex in the sense that other concepts such as *calming*, *protective*, *hectic*, *belonging*, *stability,* and *appropriateness* have been proposed [95,96].

It is possible, in this regard, to conceive of music listening in terms of *exploratory behavior* with listeners relying on listening strategies that may be quite idiosyncratic in managing attentional focus and levels of perceptual and conceptual processing of the music [97,98,99,100]. Both the search for meaningful acoustic cues—a typical timbre, a rhythmic motif, a crescendo, a trilled note, an interesting harmonic modulation, etc.—as well as the ability to make sense of broader spans of the musical flux as a kind of wayfinding in the overall mixture of musical sounds, call forth processes of *selection*, *differentiation*, *discrimination*, and *identification* [13,47]. This implies ways of listening, which can go in the direction of a search for safety or a search for novelty and challenges, somewhat related to the soundscape analogy [97,101,102]. What is meant, then, is the survey of the environment in terms of complexity, surprisingness, novelty, incongruity, mystery, and patterns, all of which can be considered to entice exploration by providing the possibility of gathering information of an environment that is complex enough to be promising, but not so complex as to be unreadable. This is related to Berlyne’s psychobiological contribution to empirical aesthetics, in which he defines aesthetic appreciation as a function of perceived arousal. Optimal arousal, in this view, is found when stimuli are in the middle between novelty and banality [103] (see also [11]). Sound environments, in this analogy, can be mildly stimulating or wildly challenging to the extent that they invite listeners to cope with novel sounds.

### 6.2. From Coping to Reward: Music and Arousal Regulation

Conceiving of music in terms of a sound environment makes it possible to come to grips with music through the mechanism of environmental appraisal. Music, in this light, can have rewarding or aversive properties, so it can be conceived as a possible stressor [99]. The avoidance of stressors and aversive stimuli, furthermore, is a basic homeostatic mechanism that is grounded in our biological dispositional machinery [62,78]. It prompts us—in the case of proper functioning—to search for stimuli within an *optimal zone of stimulation* with the frequency characteristics and acoustic levels of sounds that can be considered as beneficial rather than harmful [104,105].

Classical music mostly shows a spectrum that is roughly limited to 50–8000 Hz, but in the traditional rock music spectra, this range is extended down to 40 Hz and also upward in the higher frequencies. The possibility of producing high-level sounds, in particular in the low-frequency range, moreover, may raise questions about safety for the listener’s auditory system as well as a range of behavioral and/or physiological reactions [39]. Some dynamic features of acoustic signals such as crest factor and peak pressure values may be expected to be important eliciting factors for startle reflexes and other kinds of responsive behavior. Peak pressures in the range of 100 dB are quite common in music, and a classical symphonic orchestra playing a fortissimo may even reach 118 dB; but also sound levels during masses reach average peak levels of almost 97 dB; the values for rock groups cover a range up to 105–110 dB [106] and nowadays public address systems are even designed to deliver peak levels of 130 dB [104].

This brings us to the danger of *overstimulation* and the corresponding management of arousal. There is, in this regard, the above-mentioned psychobiological model of arousal, which states that aesthetic responses are determined by the physiological arousal we experience and is supported by research into music aesthetics that found an inverted U-shape between music preference and the complexity or arousal expressed by the music (see original research [69,103,107]. Enjoyment of music, it is stated, is optimal at intermediate arousal levels, hence the feasibility to assess music in terms of valence and arousal (see [108] for an overview). From a biological point of view, it makes sense to attune ourselves to sound environments and sonic landscapes including music that provide stimulation in the optimal arousal zone, allowing us to cultivate an ensemble of positive adaptive reactions to beneficial stressors as well as to avoid possible distress triggered by harmful stimuli or activities [109,110]. This is, in a nutshell, the definition of the concept of *eustress*, as introduced by Selye, and which he contrasted to *distress*, as the syndrome that is triggered by unspecific harmful stimuli or activities [111,112,113]. Eustress represents the pleasant stress of fulfillment while simultaneously avoiding the harmful consequences of damaging distress. It includes the properties of the stressor, which is considered to be a beneficial stressor in this case, the effort that is valued in terms of positive valence, and the effects that guarantee no damaging outcomes. Stressors, then, can be seen as beneficial when they do not exceed our capacity for maintaining or restoring our homeostasis [114].

The concept of stressor is an interesting conceptual tool to conceive of music listening in terms of coping behavior. Listeners may be “challenged” by the music, which may urge them to cope with obstacles in the sense of problem-oriented coping and to feel expansive or joyous about their struggle on the way (see [6]). The distinction between beneficial and harmful stressors, moreover, is not drastic, but there is always the biological disposition to respond to stress-inducing stimuli, which is primarily designed to provide the rapid availability of energy when needed in case of danger and stress [10]. Stressors can cause homeostatic imbalance as well as generalized stress reactions governed by the sympathetic activation of the autonomic nervous system, which can elicit physiological and hormonal changes as well as perceived disturbance and annoyance, but also cognitive impairment, sleep disturbance, and cardiovascular health conditions. Translated to the realm of music, this implies that we may conceive of the sound environment as a possible stressor, particularly with regard to high-intensity/low-frequency sound (HI/LF) and loud percussive sounds [115].

A distinction should be made, furthermore, between those stimuli that can be qualified as stressors in “objective” terms and those that are valued in terms of “subjective” appraisals. The former refers to the auditory effects of harmful stimuli that cause hearing damage and which impinge upon our auditory sense beyond deliberate and conscious control. These findings have been reported extensively and can be reduced mainly to damage the hair cells, temporal and permanent auditory threshold shifts, damage of the auditory nerve, and spiral ganglia (see [39] for an overview). However, there is also a subjective appraisal of loud sounds, which can be considered by some listeners to produce beneficial changes both in the external environment and their internal physiology and psychology [101,109].

Listeners, in this view, may become conditioned to the loudness of the stimuli, which they then begin to like and to desire [40,41], which brings us to the phenomenon of addiction to loud sounds and the problem of maladaptive listening.

### 6.3. Addiction and Maladaptive Listening

From a neurobiological point of view, music can be shown to exert powerful psychological effects through a wide range of sensory, emotional, cognitive, and motor channels, which may reflect the dynamics of coping with the sonic world. The search for these effects, however, can be “addictive” in the sense that it is no longer felt like a normal feeling. Addiction, however, must be defined in terms of costs and benefits and it can also be defined in two ways: either as positive or negative addiction. Positive addiction, as the term was coined by Glasser, refers originally to the phenomenon of “runner’s high”, a phenomenon where the brain causes endorphins and adrenaline to be released in the blood to anesthetize the pain and enhance performance. Such natural pain killers should then act as a kind of narcotic, and cause euphoria and peak experiences [116] (see also [115,117]). It is not difficult to extrapolate this to other domains beyond physical exercises. The case of music is quite typical, especially in the case of loud sounds. This is obvious in rock music and electronic music, but even classical music lovers may endorse loud sounds. Aside from the release of endorphins and adrenaline, this behavior is pushed by the search for increased emotions, which, in case of overabundance, may evolve to a kind of *maladaptive listening*, somewhat similar to the behavior exhibited by substance users [118]. Music, in fact, can be compared to acknowledged addictive substances such as alcohol, heroin, and nicotine, which has the capacity to induce rapid and potent changes in mood and level of arousal, but also to reduce negative states and the tendency to elicit the tendency of craving [119,120]. There is, however, a nonlinear relation between the search for happiness and the beneficial outcomes in the sense that this search is only beneficial up to some moderate degree. When the costs are greater than the benefits at extreme degrees, there are no longer benefits, as evidenced by markers of psychopathology, cases of happiness overdrive, mania, and dysfunctional behavior, and poorer clinical functioning [121].

In the case of loud music, a conditioning model has been proposed by Welch and Fremaux to explain why sounds at levels that can injure the ear are regarded as enjoyable by many people. This model, coined as the *CAALM model* (short for Conditioning, Adaptation, and Acculturation to Loud Music), states that the benefits associated with loud sound provide the unconditioned stimuli that people may become conditioned to enjoy over time for itself. Exposure to loud sound may cause adaptation within the auditory system to facilitate the desire for and tolerance of loud sound during leisure time, and cultural acceptance has led to an expectation of loud music at many events and celebrations, which perpetuates the conditioning [40]. The costs of acoustic overexposure, however, are high with physical injury occurring within the cochlea including the hair cells, stria vascularis, synapses, and auditory nerve fibers [122]. Furthermore, noise-induced cochlear synaptopathy and permanent loss of high-threshold nerves has been reported in young animals [86] and it has been speculated that this may be the cause of so-called “hidden hearing loss” (loss of ability to hear at higher levels while having normal hearing in quiet) in humans. Hearing loss may be associated with perceptual phenomena like tinnitus and reduced tolerance to moderate-level sounds, homeostatic changes, upregulations of synaptic gain in central auditory neurons after the loss of input of ascending fibers, enhanced auditory startle reflex, and hyperacusis [39]. It is important, therefore, to assess the patterns of damage risk with sufficient knowledge of the permissible range of possible stimulus parameters to evaluate the effects of the total physical energy delivered during exposure. This is one of the major aims of an ecologically motivated way of coping with music as part of the environmental sonic world.

## 7. Conclusions: From Reactive Behavior to Sense-Making

Listening to music is a complex process that can be explained in terms of coping behavior. It relies on levels of processing including sensory, physiological, behavioral, and cognitive ones, and depends both on hereditary and learned factors. There is no simple relationship between music’s acoustic cues and the reactions by listeners. On the other hand, there are several underlying mechanisms of coping in terms of preserving homeostatic functioning and sense-making, which point in the direction of minimizing the waste of energy and searching for optimal processing of incoming information. The development of listening strategies and listening habits are typical cases and can be considered as acquired ways of coping with the sounds. This explains the selection of music on the basis of needs in particular situations, where music can be used to modify both the internal and external environment by acting as a biological reinforcer with psychophysiological, neurochemical, and hemodynamic effects [123]. It can also be a facilitator of emotionally arousing experiences such as pleasure experiences, chills, and thrills. These feelings can trigger listeners to start an active search for acoustic cues in music, much as they would do in natural sound environments. Listening, then, is both a dispositional process, which relies on our evolutionary toolkit for coping with the world and an acquired skill for sense-making that goes beyond primitive action–reaction couplings by introducing higher-order intermediary variables between sensory input and effector reactions. As such, it calls forth a dynamic tension between bottom-up and top-down-processing of the music.
